# P-203. Incidence of Influenza in Agricultural Workers: The Agricultural Worker COVID-19 Asymptomatic and Symptomatic Transmission in the Home and Workplace (AGRI-CASA) Study, 2023-2024

**DOI:** 10.1093/ofid/ofaf695.425

**Published:** 2026-01-11

**Authors:** Molly Lamb, Daniel Carreon, Brittany Adams, Neudy C Rojop, Mirella Calvimontes, Claire Bradley, Chelsea Iwamoto, Kareen Arias, Julio del Cid-Villatoro, Daniel Vásquez, Ashley Fowlkes, Daniel Olson

**Affiliations:** Colorado School of Public Health, Aurora, Colorado; Centers for Disease Control and Prevention, Atlanta, Georgia; Centers for Disease Control and Prevention, Atlanta, Georgia; Fundacion Para La Salud Integral de los Guatemaltecos, Los Encuentros, Retalhuleu, Guatemala; Center for Human Development, Coatepeque, Retalhuleu, Guatemala; Fundación para la Salud Integral de los Guatemaltecos, Burlington, VT; Center for Disease Control and Prevention, Atlanta, Georgia; Fundacion Para La Salud Integral de los guatemaltecos, Los Encuentros, Retalhuleu, Guatemala; Fundación para la Salud Integral de los Guatemaltecos, Burlington, VT; Fundación para la Salud Integral de los Guatemaltecos, Burlington, VT; Centers for Disease Control and Prevention, Atlanta, Georgia; CU School of Medicine, Denver, Colorado

## Abstract

**Background:**

Influenza virus infections can lead to substantial productivity losses among agricultural workers essential to food security throughout the Americas including the United States, and may impact household health and economic wellbeing through transmission to family members.

**Methods:**

From January 2023 – August 2024, we estimated the burden of influenza virus infection among a cohort of 170 households in a rural, agricultural community in southwest Guatemala. Households underwent active twice weekly surveillance for influenza-like illness (ILI), defined as ≥ 1 day of fever AND either cough or sore throat. Upon respiratory symptom onset, a respiratory swab specimen was collected and tested for influenza using RT-PCR using the Roche cobas Liat instrument. Influenza vaccination status was collected at time of vaccine administration for agricultural workers, and via retrospective self-report for household members, to whom we did not directly administer influenza vaccinations.

**Results:**

Among 247 ILI cases, 112 occurred in 2023 and 135 in 2024; 95 (38%) tested positive for influenza, including 80 (84%) with influenza A and 15 (16%) with influenza B viruses. Influenza incidence in 2023 was 3.0 per 1000 persons-weeks [PW] (95% confidence interval [CI]: 2.2–4.2; influenza A 2.6 [95% CI: 1.8–3.5]; influenza B 0.5 [95% CI: 0.2–0.9]) and in 2024 3.3 per 1000 PW. (95% CI: 2.4–4.4; influenza A 2.8 [95% CI 2.0–3.7], influenza B 0.6 [95% CI 0.2–1.1]). Influenza incidence did not differ significantly by sex or age. Only 3 (3%) influenza cases received influenza vaccine in the year prior to illness onset; 92 (97%) did not. The influenza season in Guatemala peaks in early summer; most influenza cases occurred between April and July (2023: 89%, 2024: 96%) and most cases (n = 92) were unvaccinated.

**Conclusion:**

In an agricultural community with low vaccination coverage, we have described the annual burden of influenza, noted that it is largely driven by influenza A viruses, and that the influenza season peaks in the early summer months. Economic stakeholders may consider influenza vaccination programs to ensure worker health, productivity, and sustainability of food supply throughout the Americas.

**Disclosures:**

Molly Lamb, PhD, Merck: Grant/Research Support Daniel Olson, MD, Fundacion para la Salud Integral de los Guatemaltecos: Board Member|Merck: Grant/Research Support|Roche Diagnostics: Grant/Research Support

